# Influence of Acute Antipsychotic Treatment on Cardiorespiratory Coupling and Heart Rate Variability

**DOI:** 10.7759/cureus.2066

**Published:** 2018-01-15

**Authors:** Roboam R Aguirre, Mixel Z Mustafa, Alessandra Dumenigo, Steffen Schulz, Andreas Voss, Bishoy Goubran, Rhaisa Dumenigo, Marcos A Sanchez-Gonzalez

**Affiliations:** 1 Division of Clinical and Translational Research, Larkin Community Hospital; 2 Department of Psychiatry, Larkin Community Hospital; 3 Institute of Innovative Health Technologies, University of Applied Sciences Jena, Jena, Germany

**Keywords:** schizophrenia, autonomic function, cardiorespiratory coupling, respiratory rate variability, heart rate variability

## Abstract

A major contributing factor associated with increased cardiac mortality in patients with schizophrenia (SCZ) seems to be a dysfunction of the autonomic nervous system (ANS). The link between ANS dysfunction and SCZ is multifactorial, but some reports suggest that the use of antipsychotics could be implicated. This case illustrates the time course of autonomic improvement in response to antipsychotic treatment in an inpatient with SCZ in acute psychosis. To this end, we documented markers of autonomic function during hospitalization. Heart rate variability (HRV; cardiac autonomic modulation) analysis showed an increased variability over time (from Day 1 to Day 3), with strongest reaction at Day 3. The respiration analysis showed an increased respiratory variability over time (from Day 1 to Day 3) suggesting improved autonomic modulation in response to the pharmacotherapy. Cardiorespiratory coupling (CRC; surrogate of cardiorespiratory synchrony and cardiovagal modulation) showed an increasing influence of heart rate on respiration and increased from Day 1 to Day 3. The concurrent improvement of psychosis and autonomic function in response to antipsychotic treatment in this patient suggest a potential cardio protective role of antipsychotics in the acute setting. Prospective trials aimed at examining the cardiovascular implications of acute psychosis treatment in patients with schizophrenia are warranted.

## Introduction

Converging evidence suggests that patients with schizophrenia (SCZ) are at increased cardiovascular risk, which is believed to be linked to a myriad of factors such as metabolic syndrome, diabetes, and deleterious health behaviors [1, 2]. Antipsychotics, considered the mainstay therapeutic strategy for SCZ, have been documented to evoke weight gain, cardiac electrophysiological abnormalities, and adverse cardiometabolic profiles, which in turn increase cardiac risk [3, 4]. Although the precise pathophysiological mechanism linking increased cardiac risk to SCZ is not well understood, dysfunction of the autonomic nervous system (ANS) seems to be implicated [5]. Moreover, ANS dysfunction, characterized by a shift towards increased sympathetic and decreased vagal tone, has been associated with higher risk of cardiac mortality [6]. Interestingly, autonomic dysfunction is also suggested to manifest as low heart rate variability (HRV) [7] and impaired cardiorespiratory coupling (CRC) in patients with SCZ [2, 7, 8]. However, whether ANS dysfunction is a consequence of antipsychotic use or an inherent feature of SCZ remains controversial.

Some reports point out that the use of antipsychotics might act as a causative factor of autonomic dysregulation in SCZ in view of the fact that atypical antipsychotics are associated with increased risk of QT prolongation, ventricular arrhythmias, and sudden cardiac arrest [5, 8-10]. On the other hand, studies conducted in the treatment of naïve SCZ patients have shown elevated cardiovascular risk factors suggesting a potential role of psychosis and associated stress on altered cardiac function.

In addition to the heart rate (HR) changes in response to metabolic demands, the breathing rate is also constantly influenced by the emotional state [3]. In fact, the respiratory rate (RR) was found to be increased in patients with SCZ and the breathing pattern showed a pronounced association with psychopathology, including positive and negative symptoms and anxiety [3]. In this vein, aberrant cardiorespiratory coupling (CRC), or the synergy between the cardiac and the respiratory system, could also be an indicator of psychopathology. However, the impact of acute antipsychotic administration on CRC in a schizophrenic patient with acute psychosis has not been extensively documented. Accordingly, this case documents the influence of acute antipsychotic treatment on CRC and HRV and it illustrates the time course of concurrent autonomic improvement and psychosis resolution in an inpatient with SCZ.

## Case presentation

A 49-year-old Hispanic male with a past medical history of SCZ presented to the emergency room because of auditory hallucinations and aggressive behavior. The patient was admitted to the psychiatric unit with the chief complaint of a female voice telling him to hurt someone, seeing people hurting each other, and also because he couldn’t sleep well. Upon admission, his behavioral assessment findings were paranoia, withdrawal, depression, anxiety, auditory and visual hallucinations; he described seeing a woman stabbed to death by another woman at the assisted living facility where he resided, as well as seeing ghosts and a deity at his feet. During acute stay, the pharmacological agents administered upon admission were Risperdal 3 mg orally twice a day, haloperidol 5 mg/mL IM injection as needed, lorazepam 2 mg/mL IM injection as needed, and citalopram 40 mg orally daily.

In the inpatient unit, a monitoring biosignal device, a heart/respiration rate monitor (Zephyr Biopatch, Annapolis, MD) was placed on the chest of the patient overnight and the following day in the morning but removed at around 9:00 am. Thereafter, the physiological monitoring was repeated for two more consecutive days. Clinical improvement was observed on Day 2 after medication treatment began; the patient was cooperative and denied hallucinations and homicidal ideations. His attention, thought content, insight, and judgment also improved; however, his thought process was linear with a slow flow of thought.

**Figure 1:**
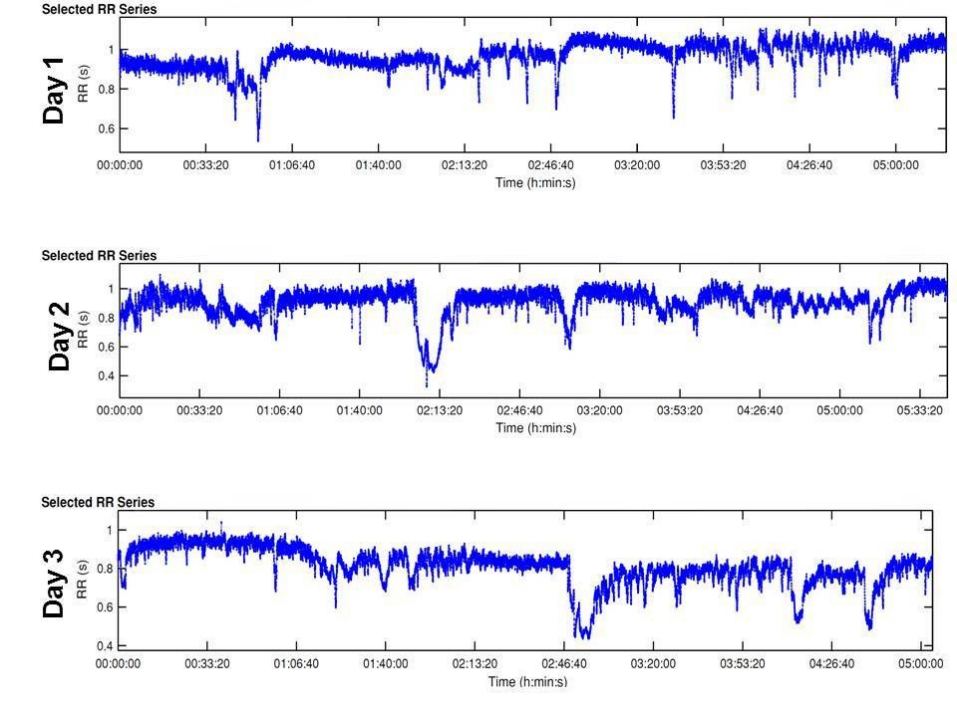
Tachograms of beat-to-beat intervals during nocturnal monitoring These strip segments are samples of overnight beat-to-beat variation of the test subject throughout the three days. The blue tracing shows gradual decline throughout the days demonstrating increasing variability. The complete tracing was analyzed to obtain the frequency and time domains of HRV in each day to compare autonomic regulation. HRV: heart rate variability.

For HRV, R-R intervals obtained on the monitoring device were subsequently analyzed using Kubios HRV® (Kubios, Kuopio, Finland) (see Figure 1).

**Figure 2:**
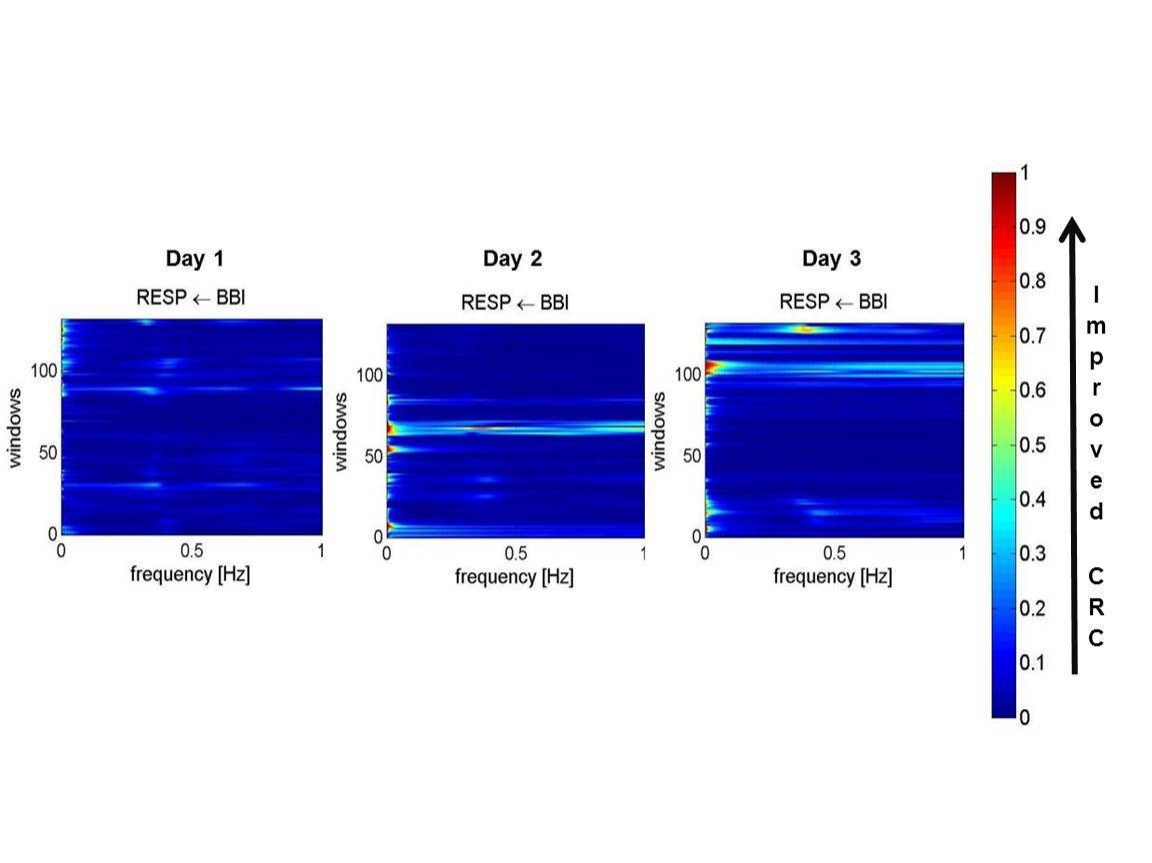
Plots for cardiorespiratory coupling analyses (CRC) Arrows indicate the causal coupling direction from one time series to another time series, e.g., BBI←RESP, indicating the causal link from RESP to BBI. The coupling strength ranges from blue (no coupling, 0) to red (maximum coupling, 1) with Area_BBI→RESP (Day 1 = 0.05; Day 2 = 0.08; Day 3 = 0.10). BBI: beat-to-beat intervals, RESP: time intervals between consecutive breathing cycles.

The time series between the times of 9:00 p.m. to 3:00 a.m. (~five hours duration for each day) results showed that the HRV basic heart rate (HR, meanNN = mean value of NN intervals) did not change over time, Day 2 showed the most prominent change in HRV (increased). Day 3 showed partly higher values of HRV in comparison to Day 1; in general, the variability standard deviation of normal R-R-intervals (sdNN) increased over time from Day 1 to Day 3, with the strongest reaction at Day 2 (Day 1 = 0.06 ms; Day 2 = 0.12 ms; Day 3 = 0.11 ms). With regard to respiration, the respiration mean rate (meanNN) did not change over time; respiratory variability (RESPV; sdNN) increased over time from Day 1 to Day 3 (day 1 = 0.10 ms; Day 2 = 0.18 ms; Day 3 = 0.19 ms). In CRC (normalized short time partial directed coherence (NSTPDC), 120 samples window length) the influence of heart rate (BBI) on respiration (RESP) increased from Day 1 to Day 3  (Area_BBI→RESP : Day 1 = 0.05; Day 2 = 0.08; Day 3 = 0.10) and the influence of RESP on BBI decreased from Day 1 to Day 3 (Area_RESP→BBI: Day 1=0.23; Day 2=0.18; Day 3=0.13). Considering the coupling direction, the normalized factor (NF) values showed an increase from Day 1 to Day 3. Day 1 (NF~ -1.3) pointed to a bidirectional coupling, with a driver-responder relationship from RESP→BBI, Day Two (NF~-0.8) pointed to a weak bidirectional coupling with a driver-responder relationship from RESP→BBI, and Day 3 (NF~-0.34) pointed to an equal influence in both directions (see Figure 2) suggesting a more balanced autonomic cardiorespiratory modulation in response to the antipsychotic treatment where respiration still acts as the driver (respiratory sinus arrhythmia). The HRV analysis showed an increased variability and improved cardiovagal modulation over time (from Day 1 to Day 3), with strongest reaction at Day 2.

The respiration analysis showed an increased respiratory variability over time (from Day 1 to Day 3) suggesting improved autonomic modulation in response to the pharmacotherapy. The CRC showed that the influence of heart rate on respiration increased from Day 1 to Day 3. The numeric sequence test, used for testing attention, was '4' (higher values are associated with better attention) during Day 1. The attention span increased during Day 2 and Day 3 with scores of '8' and '9', respectively. Upon discharge on Day 4, improvement was observed in the patient's general psychiatric condition; he was calm, cooperative, and denied visual and auditory hallucinations.

## Discussion

Recently, the interest in understanding cardiac autonomic function in patients with SCZ treated with antipsychotics has been increasing. In this case, we sought to document cardiac autonomic regulation (HRV, CRC, respiratory rate variability (RRV)) effects and the clinical response to antipsychotic treatment in a schizophrenic patient with acute psychosis. The results of this case demonstrate that in a patient with SCZ, the effects between decompensated psychosis and autonomic dysregulation improve with the addition of appropriate antipsychotic pharmacotherapy during a three-day hospital stay. Even though the cardiac effects of antipsychotics are still nonconclusive [4, 5], our case suggests that medication as acute management of psychosis does not induce cardiac dysregulation at least acutely. Moreover, the improvement in CRC, evidenced in conjunction with improved mental status, provides a potential biomarker that could be used to monitor psychotic states in patients with SCZ. This could be easily and readily available via convenient, noninvasive wearable technologies. The notion of antipsychotic effects on cardiovascular and metabolic dysfunction suggests the need for further investigation related to the overlapping effects of cardiovascular and metabolic conditions and psychosis.

## Conclusions

Risk evaluation is essential when developing treatment plans for patients in general. Cardiovascular risk factors are considered in the management of patients with SCZ using antipsychotics, at least in part, owing to autonomic nervous system dysfunction. Cardiac autonomic dysregulation effects in patients with acute psychotic episodes appear to improve when patients are treated with antipsychotics. This case has shown that acute psychotic symptoms decrease with antipsychotic therapy in a patient admitted to the hospital and suggests that medication as acute management of psychosis does not induce cardiac dysregulation and improves cardiorespiratory coupling and attention. The concurrent improvement of psychosis and autonomic function in response to antipsychotic treatment in this patient suggests a potential cardio protective role of antipsychotics in the acute setting. Prospective trials aimed at examining the cardiovascular implications of acute psychosis treatment in patients with schizophrenia are warranted.
